# Arsenic trioxide, a potent inhibitor of NF-κB, abrogates allergen-induced airway hyperresponsiveness and inflammation

**DOI:** 10.1186/1465-9921-7-146

**Published:** 2006-12-20

**Authors:** Lin-Fu Zhou, Yi Zhu, Xue-Fan Cui, Wei-Ping Xie, Ai-Hua Hu, Kai-Sheng Yin

**Affiliations:** 1 Department of Respiratory Medicine, The First Affiliated Hospital, Nanjing Medical University, Nanjing, China; 2 Global Health Programs, University of Pennsylvania School of Medicine, Philadelphia, USA; 3 Division of Pulmonary Medicine, Joseph Stokes Jr. Research Institute, The Children's Hospital of Philadelphia, University of Pennsylvania School of Medicine, Philadelphia, USA

## Abstract

**Background:**

Overactivation of nuclear factor κB (NF-κB) orchestrates airway eosinophilia, but does not dampen airway hyperresponsiveness in asthma. NF-κB repression by arsenic trioxide (As_2_O_3_) contributes to apoptosis of eosinophils (EOS) in airways. Here we provide evidence that As_2_O_3 _abrogates allergen (OVA)-induced airway eosinophilia by modulating the expression of IκBα, an NF-κB inhibitory protein, and decreases the airway hyperresponsiveness.

**Methods:**

Using a murine model of asthma, the airway hyperresponsiveness was conducted by barometric whole-body plethysmography. Airway eosinophilia, OVA-specific IgE in serum, and chemokine eotaxin and RANTES (regulated upon activation, normal T cell expressed and secreted) in bronchoalveolar lavage fluid were measured by lung histology, Diff-Quick staining, and ELISA. Chemokine-induced EOS chemotactic activity was evaluated using EOS chemotaxis assay. Electrophoretic mobility shift assay and Western blot analysis were performed to assess pulmonary NF-κB activation and IκBα expression, respectively.

**Results:**

As_2_O_3 _attenuated the allergen-induced serum IgE, chemokine expression of eotaxin and RANTES, and the EOS recruitment in bronchoalveolar lavage fluid, which is associated with an increased IκBα expression as well as a decreased NF-κB activation. Also, As_2_O_3 _suppressed the chemotaxis of EOS dose-dependently *in vitro*. Additionally, As_2_O_3 _significantly ameliorated the allergen-driven airway hyperresponsiveness, the cardinal feature underlying asthma.

**Conclusion:**

These findings demonstrate an essential role of NF-κB in airway eosinophilia, and illustrate a potential dissociation between airway inflammation and hyperresponsiveness. As_2_O_3 _likely exerts its broad anti-inflammatory effects by suppression of NF-κB activation through augmentation of IκBα expression in asthma.

## Background

Asthma is now accepted as a T-helper type 2 (Th2) lymphocyte-mediated chronic inflammatory disorder, characterized by airway eosinophilia and airway hyperresponsiveness (AHR) [1]. Eosinophils (EOS) appear to play a crucial role in the ongoing inflammation due to either an impaired clearance or a delayed apoptosis in the airways, where accumulation of a number of EOS cytotoxic proteins including major basic protein, cationic proteins and peroxidase could occur [2]. Existing data support the notion that morphologic changes in airway tissue to the development and severity of AHR in asthma correlates with the presence of activated airway inflammatory cells, in particular EOS [3].

The molecular regulatory pathways in induction of chronic cytokine expression and recruitment/activation of inflammatory cells in asthma remain elusive. However, there is growing recognition that these processes involve increased transcription of inflammatory genes via transcription factors [4]. One such transcription factor, nuclear factor κB (NF-κB), is abundant of p50 (NF-κB1)/p65 (RelA) heterodimer. In a latent state, NF-κB is sequestered as an inactive trimer by complexing with IκBα, a 37 kDa inhibitory protein, which promotes cytoplasmic retention and maintains a low basal transcriptional activity. IκBα consists of an N-terminal domain containing specific phosphorylation sites, five ankyrin repeat sequences, and a C-terminal domain of Pro-Glu-Ser-Thr polypeptides [5]. Upon stimulation, IκBα is phosphorylated by the IκB kinase, ubiquitinated and degraded through the 26S proteasome pathway [6]. Subsequently, the nuclear localization sequence of NF-κB is unmasked to allow its translocation into the nucleus, where it binds to DNA and initiates transcription of a wide range of NF-κB-dependent genes in association with immune and inflammatory responses [7].

Arsenic compound has long been considered as a protoplasmic poison that can bind to human sulfydryl-containing proteins with high affinity. Arsenic trioxide (As_2_O_3_), extracted from arsenic compound, is a powerful ancient medication for a variety of ailments with the principle of "using a toxic against another toxic" in traditional Chinese medicine. Strikingly, As_2_O_3 _treatment in a regime of 10 mg/d of intravenous infusion for 28 to 60 days is effective in patients with acute promyelocytic leukemia (APL) without viable toxicity in refractory to the all-trans retinoic acid (ATRA) and the conventional chemotherapy by inducing apoptosis of APL cells [8]. Many studies have demonstrated that NF-κB overactivation underlines the chronicity of airway inflammation characteristic of asthma [9-12]. Recently, we have reported that As_2_O_3_-mediated NF-κB repression in airways facilitated EOS apoptosis in a dose-dependent manner, contributing to the resolution of airway eosinophilic inflammation [13]. In this study, we investigated the effects of As_2_O_3 _on allergen-induced AHR and NF-κB-mediated airway inflammation in a murine model of asthma. Our data indicate that inhibition of NF-κB activation through induction of IκBα expression may account for the broad anti-inflammatory action of As_2_O_3_.

## Methods

### Asthma modeling

Specified pathogen-free female BALB/c mice, aged 6 to 8 weeks, were provided by the Chinese Academy of Medical Sciences (Beijing, China). The animal experiment was approved by Nanjing Medical University according to the guidelines of the Institutional Animal Care and Use Committee. A murine asthma model was established as described previously [14] with minor modifications.

On days 0 and 7, mice received intraperitoneal injection of 20 μg of chicken ovalbumin (OVA, Grade V, Sigma-Aldrich, St. Louis, MO) adsorbed to 20 mg of aluminum hydroperoxide gel (Pierce, Rockford, IL). On days 14, mice were randomized to receive aerosol challenge with either 6% OVA in phosphate-buffered saline (PBS) or PBS alone via a nebula (1–5 μM particles, Bohringer Ingelheim, Germany) for 40 min per day up to 7 days. During the treatment period, As_2_O_3 _(Yida Pharmaceutics, Harbin, China) at dose of 0.5–4.5 mg/kg, dexamethasone (Dex, Phoenix Pharmaceutics, Belmont, CA) at dose of 2.5 mg/kg or PBS alone was injected into the peritoneum 30 min before each airway challenge. After the last aerosol exposure, mice were sacrificed at designated timepoints.

### Airway physiology

Baseline resistance and AHR induced by nebulized methacholine (Sigma-Aldrich, St. Louis, MO) at dose of 12.5–100 mg/ml in conscious unrestrained-mice were assessed using barometric whole-body plethysmography (Buxco Electronics Inc., Troy, NY) as described previously [15]. Airway resistance is expressed as: *P*_enh _= [(*T*_e_/0.3 *T*_r_)-1] × [2 *P*_ef_/3 *P*_if_], where *P*_enh _= enhanced pause, *T*_e _= expiratory time (sec), *T*_r _= relaxation time (sec), *P*_ef _= peak expiratory flow (ml/sec), and *P*_if _= peak inspiratory flow (ml/sec).

### Bronchoalveolar lavage

Four hours after the last airway challenge, mice underwent euthanasia and were cannulated in the trachea. The lungs were washed twice with 1 ml aliquots of PBS to collect the bronchoalveolar lavage fluid (BALF). Subsequently, the lungs were removed, quickly frozen in liquid nitrogen, and stored at -70°C. Additionally, the lungs were collected at 1, 12, and 24 hrs post the last airway challenge to study the kinetics of pulmonary NF-κB activation.

### Lung histology

Paraffin embedded lung sections (5 μm) collected 24 hrs after airway challenge were stained with hemotoxylin & eosin (Sigma-Aldrich, St. Louis, MO) for examination of histology.

### Diff-Quick staining

Diff-Quick staining is a modified Wright's staining [16]. Centrifuged at 300 × *g *for 10 min, the pelleted cells of BALF were suspended in a serum-free RPMI 1640 medium. The cell viability, evaluated by the trypan blue exclusion method, was over 95%. Total and differential cell counts were enumerated on cytospins (Thermo Shandon, Pittsburgh, PA) in compliance with the Diff-Quick staining profile (Merck, Germany) by counting at least 200 to 500 cells in cross-section.

### Enzyme-linked immunosorbant assay (ELISA)

Serum levels of OVA-specific immunoglobulin E (IgE) were analyzed by ELISA using samples collected 24 hrs after the last OVA challenge. Briefly, 96-well plates were coated with either purified anti-mouse IgE (5 μg/ml, BD PharMingen, San Diego, CA) or OVA (100 μg/ml). After addition of serum samples, OVA-specific IgE was detected using horseradish peroxidase (HRP)-conjugated sheep anti-IgG (Calbiochem, La Jolla, CA). Arbitrary units (AU) were calculated according to OD_50 _of the standard curve.

Murine chemokines, eotaxin and RANTES (regulated upon activation, normal T cell expressed and secreted), in the BALF samples were measured by utilizing paired antibodies following the manufacturer's recommendations. The ELISA kits were purchased from R&D Systems (Minneapolis, MN) with a minimum detectable levels of 3 and 5 pg/ml for eotaxin and RANTES, respectively.

### EOS chemotaxis assay (ECA)

Interleukin (IL)-5 transgenic mice (CBA/CaH-TnN) were provided by the Institute of Chemistry and Cell Biology, Chinese Academy of Sciences (Shanghai, China). EOS (~98% purity) were derived from spleen of IL-5 transgenic mice with depletion of B, T, and antigen-presenting cells using anti-B220, anti-CD4, anti-CD8 and anti-class II, as well as rat anti-mouse Ig-conjugated magnetic beads (Miltenyi Biotec, Auburn, CA) as described previously [17]. EOS were seeded at 5 × 10^4 ^density in triplicate and preincubated for 15 min at room temperature with 0.25–2 μM of As_2_O_3 _prior to chemotaxis measurement.

Chemotaxis was assessed in 48-well micro-Boyden chambers using polyvinylpyrrolidone-free polycarbonate membranes (NeuroProbe, Bethesda, MD). Cell suspension and diluted chemokines of eotaxin or RANTES (PeproTech, London, UK) were added into the chamber with RPMI 1640 containing 25 mM N-2-hydroxyethylpiperazine-N'-2-ethanesulfonic acids (HEPES, pH 7.4) and 0.05% bovine serum albumin. The plates were incubated for 60 min at 37°C under 5% CO_2_. The migrated cells were counted in five randomly selected high-power fields (magnification was × 1,000). Spontaneous migration was evaluated in the absence of chemoattractant.

### Extraction of nuclear and total proteins

Nuclear and total proteins of lung tissue were collected as described previously [18]. Briefly, aliquots of liquid nitrogen-frozen tissue were pulverized and lysed in 200 μl of cold Buffer A [10 mM Tris-HCl (pH7.5), 150 mM NaCl, 1.5 mM MgCl_2_, 0.65% Nonidet P-40, 0.5 mM phenylmethylsulfonyl fluoride (PMSF) and 0.5 mM dithiothreitol (DTT)] for 3 min. After centrifugation at 10,000 × *g *for 1 min at 4°C, the nuclear pellets were extracted with 20 μl of Buffer B [20 mM HEPES (pH7.9), 1.5 mM MgCl_2_, 420 mM NaCl, 0.5 mM DTT, 0.2 mM ethylenediaminetetraacetic acid (EDTA), 0.5 mM PMSF and 25% glycerol] for 30 min with intermittent mixing on ice. The supernatant containing nuclear proteins was collected by centrifugation at 12,000 × *g *for 5 min.

The total proteins were prepared by addition of Buffer A to the lung powder and subjected to two freeze/thaw cycles to fracture the nuclear membranes. After centrifugation, the supernatant was collected. The nuclear and total proteins were quantitated using the Bradford assay (BioRad, Hercules, CA), aliquoted and stored at -70°C until use.

### Electrophoretic mobility shift assay (EMSA)

EMSA analysis was performed using a commercial kit (Promega, Madison, WI). Double-stranded oligonucleotide probe (5'-AGTTGAGGGGACTTTCCCAGGC-3') containing a consensus NF-κB sequence (underlined) was end-labelled with [γ-^32^P]-adenosine triphosphate (Furui Biotechnology, Beijing, China) by T4 polynucleotide kinase and purified by chromatography. The binding reaction was conducted in a final volume of 20 μl containing 5 μg of nuclear proteins and 30 fmol of ^32^P-labelled oligonucleotide probe. Protein-DNA complexes were separated by electrophoresis on a 5% native polyacrylamide gel (37:1 acrylamide:bis-acrylamide) in a 0.5 × Tris-borate-EDTA running buffer. The dried gel was exposed to PhosphorImager (Molecular Dynamics) using ImageQuant software (Amersham Life Science, Arlington Heights, IL).

For competition assay, a 100-fold excess of unlabelled NF-κB or activator protein 1 (AP-1) oligonucleotide probe was added to the reaction mixture 10 min before addition of the labelled probe. For supershift assay, a 0.5 μg of anti-p50 or anti-p65 antibody (Santa Cruz Biotechnology, Santa Cruz, CA) was added to the reaction mixture prior to the labelled probe for 30 min.

### Western blot analysis

Denatured samples (100 μg of total proteins) were fractionated by 10% sodium dodecyl sulfate polyacrylamide gel eletrophoresis (SDS-PAGE) and transferred to nitrocellulose membranes. Blots were blocked with 5% milk containing 1 × TBST [40 mM Tris-HCl (pH7.6), 300 mM NaCl and 0.1% Tween-20] at 4°C overnight. Thereafter the blot was probed with primary antibodies of anti-IκBα (1:1,000 dilution) or anti-β-actin antibody (1:800 dilution) for 1 hr. After an HRP-conjugated goat anti-rabbit IgG (1:5,000 dilution, Santa Cruz Biotechnology, Santa Cruz, CA) incubation, the immunoblots were visualized by an enhanced chemiluminescence (ECL) kit (Pierce, Rockford, IL) according to the manufacture's instructions.

### Data analysis

Statistical analysis was performed by one-way analysis of variance (ANOVA) and *q *test with SPSS 11.0 software package (SPSS Inc., Chicago, IL). The negative relationship was evaluated by *Pearson *correlation analysis. Data were expressed as mean ± SEM, and p < 0.05 was considered statistically significant.

## Results

### Attenuation of airway EOS recruitment by As_2_O_3_

**Figure 1:**
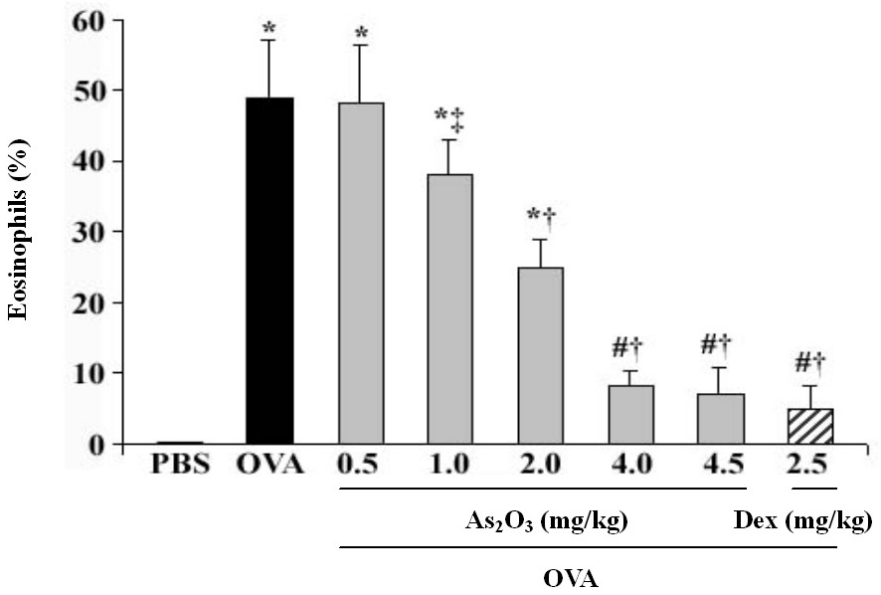
**As_2_O_3 _decreases EOS recruitment in BALF in a dose-dependent manner**. Intraperitoneal administration of OVA-challenged mice with As_2_O_3 _(0.5–4.5 mg/kg) reduced the EOS in BALF, in which both 4, 4.5 mg/kg of As_2_O_3 _and 2.5 mg/kg of Dex achieved the similar anti-inflammatory effects. BALF EOS, stained with Diff-Quick solution, were counted using a hematocytometer, and expressed as a percentage in total leukocytes. Data represent the mean ± SEM of four separate experiments (n = 6 per group). ^# ^p < 0.05, *p < 0.01, *vs *the control mice; ^‡ ^p < 0.05, ^† ^p < 0.01, *vs *the OVA-challenged mice.

**Figure 2:**
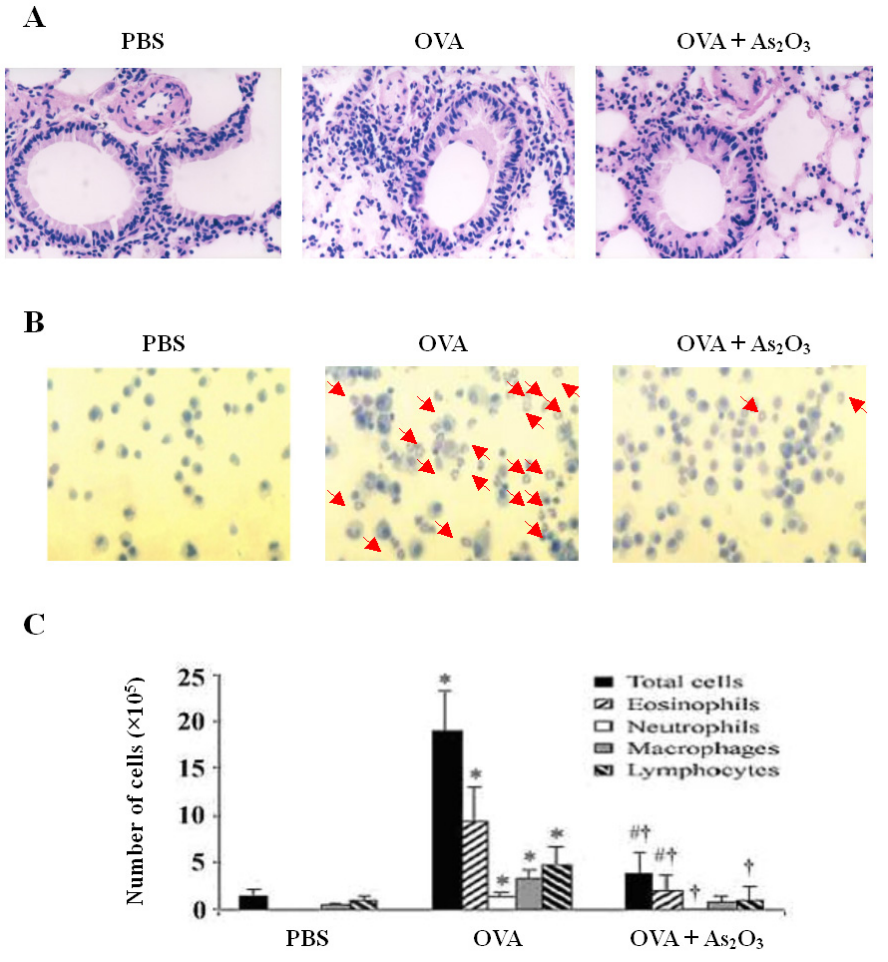
**As_2_O_3 _markedly ameliorates allergic airway inflammation**. (A) Lung tissues of naïve mice, untreated OVA-challenged mice, and OVA-challenged mice treated with 4 mg/kg of As_2_O_3 _were subjected to histological analysis by staining with hematoxylin & eosin. Magnification was × 400. (B) BALF was collected 24 hrs after the final OVA challenge, and stained with Diff-Quick for microscopic detection of EOS dyed in orangeophil red with cytoplasmic acidophil granules (arrows). Magnification was × 200. (C) Total and differential cell counts in BALF are plotted for each group. Data represent the mean ± SEM of three independent experiments (n = 6 per group). ^# ^p < 0.05, * p < 0.01, *vs *the control mice; ^† ^p < 0.01, *vs *the OVA-challenged mice.

OVA-challenged mice in response to 0.5–4.5 mg/kg of As_2_O_3 _reduced the number of EOS in BALF in a dose-dependent manner (Fig. 1). Since the anti-inflammatory effects of As_2_O_3 _were similar at the doses of 4 and 4.5 mg/kg, and it was comparable to the effect of 2.5 mg/kg of Dex (p > 0.05), the 4 mg/kg of As_2_O_3 _was herein chosen as the effective dosage in the rest of experiments. This dosage was also proved to be relatively safe based on our previous experiments [13,14]. Histological analysis of the OVA-challenged mice lung revealed an enhanced airway eosinophilia as compared to the naïve control mice that were treated with PBS (Fig. 2A). Conversely, pretreatment of As_2_O_3 _protected mice from developing the allergen-induced peribronchial inflammation (Fig. 2A). Examination of BALF collected from mice at 24 hrs after OVA challenge showed a marked influx of inflammatory cells into the airways, including EOS, lymphocytes, macrophages and neutrophils (Fig. 2B–C). The increased EOS in the BALF was correlated with an increase of EOS recruitment by the Diff-Quick analysis in OVA-challenged mice (Fig. 2B). The number of EOS in BALF from naïve mice was less than 1%, whereas that of OVA-challenged mice was about 49% (p < 0.01). Pretreatment of As_2_O_3_ dramatically attenuated the airway eosinophilia in the OVA-challenged mice (p < 0.01; Fig. 2A–C; Table 1).

**Table 1:** Effect of As_2_O_3 _on EOS recruitment in BALF (%), pulmonary NF-κB activity (relative intensity units) and IκBα expression (IκBα/β-actin).

Asthma						
	Control					As_2_O_3_
	4 hrs	1 hr	4 hrs	12 hrs	24 hrs	4 hrs
EOS	0.56 ± 0.22	5.08 ± 1.37*	11.12 ± 1.93*	20.25 ± 2.99*	48.72 ± 5.38*	4.69 ± 1.21*^†^
NF-κB	51.47 ± 4.53	162.31 ± 9.46*	255.74 ± 11.10*	127.59 ± 8.72*	80.97 ± 6.15^#^	75.80 ± 9.33*^†^
IκBα	0.80 ± 0.25	0.45 ± 0.04*	0.23 ± 0.10*	0.36 ± 0.03*	0.54 ± 0.07^#^	1.56 ± 0.34*^†^

Data represent the mean ± SEM of four independent experiments (n = 6 per group). ^# ^p < 0.05, * p < 0.01, *vs *the control mice; ^† ^p < 0.01, *vs *the OVA-challenged mice at 4 hrs.

### Amelioration of AHR by As_2_O_3_

**Figure 3:**
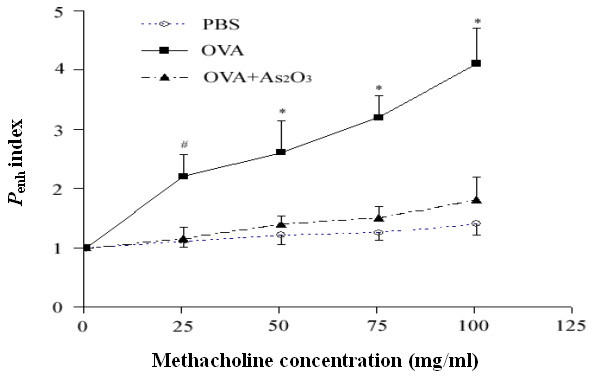
**As_2_O_3 _prohibits allergen-induced AHR**. Mice were placed in whole-body plethysmographs and underwent varying methacholine challenge 24 hrs after the last airway challenge of OVA or PBS. The OVA-challenged mice exhibited remarkable bronchial reactivity to inhaled methacholine, compared with control mice or mice challenged with OVA in the presence of 4 mg/kg of As_2_O_3_. Data represent the mean ± SEM of four independent experiments (n = 5 per group). ^# ^p < 0.05, * p < 0.01, *vs *the control or OVA-challenged mice.

*P*_enh_, relative to the measured airway resistance, was obtained as an index and was normalized to the postsaline – *P*_enh_. This readout was used as a measure of AHR. Mice previously sensitized and challenged with OVA developed a dose-dependent methacholine-induced bronchospasm as compared to the naïve mice that were treated with PBS. As_2_O_3 _treatment significantly reduced the effect (p < 0.01; Fig. 3).

### Reduction of serum IgE and BALF chemokines by As_2_O_3_

**Figure 4:**
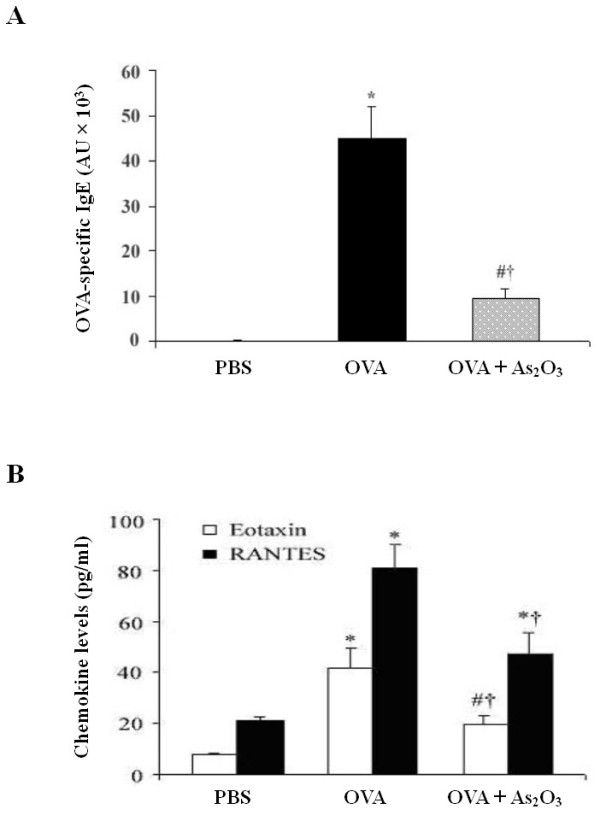
**As_2_O_3 _alleviates OVA-specific IgE in serum and eotaxin and RANTES in BALF of allergen-sensitized mice**. Serum and BALF were collected 24 hrs after the last OVA challenge. Levels of (A) OVA-specific IgE in serum and (B) chemokine eotaxin and RANTES in BALF were analyzed by ELISA. Data represent the mean ± SEM of three independent experiments (n = 6 per group). ^# ^p < 0.05, * p < 0.01, *vs *the control mice; ^† ^p < 0.01, *vs *the OVA-challenged mice.

IgE can augment allergic airway responses in a high affinity receptor-dependent manner. Serum levels of OVA-specific IgE were elevated in OVA-challenged mice compared with the naïve control mice (p < 0.01), whereas pretreatment with As_2_O_3 _resulted in a 4.8-fold decrease to the levels of the OVA mice (p < 0.01; Fig. 4A). Eotaxin and RANTES play a critical role in inducing chemotaxis of EOS [19]. ELISA analysis showed that levels of eotaxin and RANTES in BALF were markedly increased in OVA-challenged mice in comparison with the control mice (p < 0.01). However, these chemokine levels were largely reduced by pretreatment with As_2_O_3 _(p < 0.05 or 0.01; Fig. 4B).

### Ablation of EOS chemotaxis by As_2_O_3_

**Figure 5:**
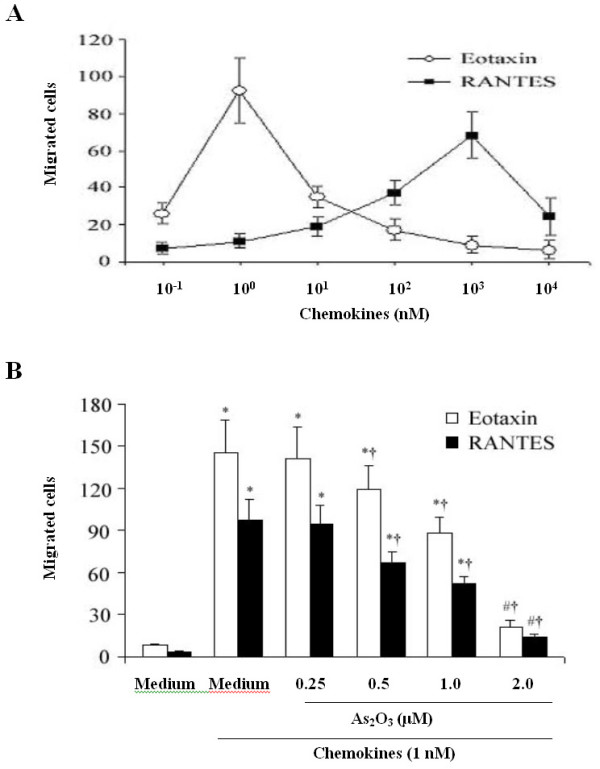
**As_2_O_3 _ablates EOS chemotaxis**. (A) Eotaxin and RANTES induced chemotaxis of EOS, in which eotaxin was more potent than RANTES. The numbers of migrating cells per five high-power fields (magnification was × 1,000) are shown. (B) Pretreatment of EOS with As_2_O_3 _15 min before transferring to the chemotaxis chamber greatly suppressed the eotaxin or RANTES-induced migration in a dose-dependent manner. Data represent the mean ± SEM of three independent experiments (n = 5 per group). ^# ^p < 0.05, * p < 0.01, *vs *the control (medium alone); ^† ^p < 0.01, *vs *the prestimulation with medium plus stimulation with 1 nM of chemokines.

Eotaxin and RANTES with respective concentrations of 1 (10^0^) and 10^3 ^nM reached a maximal chemotaxis response indicating that eotaxin is a more active chemotaxin to EOS than RANTES (Fig. 5A). As_2_O_3 _significantly inhibited the EOS chemotaxis mediated by eotaxin or RANTES in a dose-dependent manner (p < 0.05 or 0.01; Fig. 5B).

### Inhibition of pulmonary NF-κB activation by As_2_O_3_

**Figure 6:**
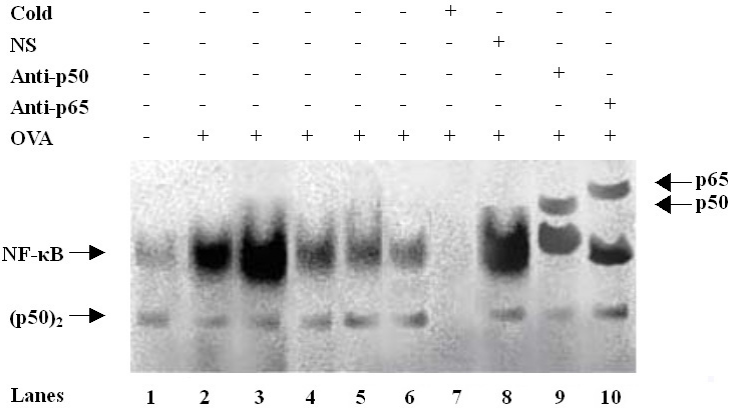
**As_2_O_3 _inhibits pulmonary NF-κB activation in OVA-sensitized and challenged mice**. Nuclear extracts of lung tissue were prepared and subjected to EMSA analysis of NF-κB activity. *Lane 1*: Naïve control mice; *Lanes 2–5*: OVA-sensitized mice 1, 4, 12, and 24 hrs after the final OVA challenge; *Lane 6*: OVA-sensitized mice treated with As_2_O_3 _4 hrs after the final OVA challenge;* Lanes 7–8*: Specific (cold) and nonspecific (NS) competition; *Lanes 9–10*: Supershifts of p50 and p65. Nuclear extracts of lanes 7 to 10 were derived from those of lane 3. Free DNA probe is not shown. The arrows indicate the specific NF-κB-DNA complexes, p50 dimer, and supershifts, respectively. One of four independent experiments is shown.

The OVA challenged mice showed a sharp increase in the pulmonary DNA binding activity of NF-κB at various timepoints as compared to the unchallenged mice lung. Indeed, NF-κB activity was increased within 1 hr (p < 0.01), peaked at 4 hrs (p < 0.01), and decreased by 12 (p < 0.01) to 24 hrs (p < 0.05). This effect of OVA challenge was clearly ameliorated by pretreatment with As_2_O_3 _(p < 0.01; Fig. 6, lane 6 as compared to lane 3; Table 1). In the competition assay, addition of 100-fold excess of unlabelled NF-κB, but not AP-1, oligonucleotide probe competed away the NF-κB-DNA complexes, verifying the specificity of NF-κB binding. In the supershift assay, addition of antibodies against p50 and p65 resulted in retardation of supershifted bands, with reciprocal decreases in the intensity of the NF-κB bands, confirming the classic subunits of NF-κB heterodimer (Fig. 6).

### Augmentation of pulmonary IκBα expression by As_2_O_3_

**Figure 7:**
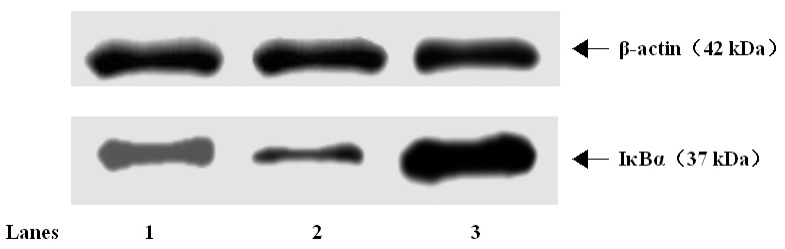
**As_2_O_3 _augments pulmonary IκBα expression in OVA-sensitized and challenged mice**. Total proteins of lung tissue were extracted 4 hrs after the final OVA challenge, and subjected to Western blot analysis of IκBα. β-Actin was utilized as the standard control. *Lane 1*: Naïve control mice; *Lane 2*: OVA-sensitized and challenged mice; *Lane 3*: OVA-sensitized and challenged mice treated with 4 mg/kg of As_2_O_3_. The positions of molecular size standards (in kDa) are indicated by arrows. One of three separate experiments is shown.

The pulmonary IκBα expression in the lung lysate was relatively decreased in OVA-challenged mice (p < 0.01; Fig. 7; Table 1) compared to the control lung. In contrast, pretreatment of As_2_O_3 _accumulated the pulmonary IκBα (p < 0.01). Furthermore, there was a tight negative correlation between EOS recruitment in the BALF or the pulmonary NF-κB activation and IκBα expression (*r *= -0.82 and -0.94, respectively; p < 0.01).

## Discussion

Multiple upstream signal events converge on the NF-κB-inducing kinase (NIK) [20]. Activation of NIK results in phosphorylation of IκB kinases, which render the phosphorylation of IκBα at N-terminal serines 32 and 36 (Ser32 and Ser36) residues, leading to a proteolytic degradation of IκBα. Consequently, the activated NF-κB translocates to the nucleus, where it bonds to specific κB sites to facilitate the transcription of target genes. This results in expression of numerous pro-inflammatory cytokines, chemokines and adhesion molecules [21]. These pro-inflammatory mediators are essential in the recruitment of airway inflammatory cells, including EOS and CD4^+ ^T lymphocytes, which in turn secret Th2 cytokines [22]. Therefore, NF-κB repression in airways via suppression of IκBα degradation or augmentation of IκBα synthesis would decrease the transcription of a myriad of NF-κB-dependent genes. This strategy proved to be more effective than that of blocking a single downstream inflammatory or an immune gene among the inflammatory cascade [23,24].

Several lines of evidence suggest a central role of NF-κB in the pathogenesis of asthma. Activated NF-κB has been identified in sputum-induced macrophages and bronchial biopsy specimens of asthmatic patients [25]. Agents such as allergens, ozone and viral infections, which are associated with exacerbation of asthma, stimulate activation of NF-κB [26]. As the major effective treatment for asthma, glucocorticoids are potent blockers of NF-κB activation [27]. Furthermore, mice lacking the NF-κB subunits p50 or c-Rel develop less airway inflammation upon antigen challenge [28]. Nevertheless, NF-κB activation orchestrates allergen-induced inflammation and subsequent adaptive responses, but does not appear to modulate AHR, the cardinal feature that underlies asthma, signifying a potential dissociation between airway inflammation and AHR [29]. Clearly, additional airway signaling pathways activated, residual NF-κB activity or other inflammatory processes may be responsible for the AHR. Alternatively, events localized more distally within the alveolar compartments, such as microvasculature leakage of macromolecules, alveolar injury or surfactant dysfunction might dominate the genesis of AHR [30-32].

As_2_O_3 _(1–2 μM) induces the apoptosis in t (15;17) APL cell line NB4 *in vitro *and in APL patients without significant myelosuppression *in vivo *[8]. We and others have confirmed that inhibition of NF-κB was essential to arsenic-induced apoptosis [13,33]. In this report, despite a decreased serum OVA-specific IgE production, we demonstrated an inhibitory effect of As_2_O_3 _on EOS recruitment from OVA-challenged BALF, in agreement with our previous observation that As_2_O_3 _promoted EOS apoptosis in the airway eosinophilic inflammation [13]. Additionally, both eotaxin and RANTES, downstream genes of NF-κB, demonstrated potent chemoattractants to EOS and Th2 lymphocytes [34]. Presumably, the ablation of airway eosinophilia by As_2_O_3 _results from a collective effects of NF-κB inhibition such as a reduced specific IgE secretion, chemokine expression and Th2 cytokine production as well as an altered eosinophilic cytoskeletal rearrangement [35,36]. Overall, As_2_O_3 _might exert its multiple anti-inflammatory action through augmentation of IκBα expression and suppression of NF-κB activation in the airways. This is partially in accordance with the therapeutic role of glucocorticoid-mediated NF-κB repression in asthma [37,38]. Interestingly, in this model of asthma, As_2_O_3 _abrogated both allergic airway inflammation and AHR in contrast with the previous report [29], suggesting a specific effect of As_2_O_3 _besides NF-κB suppression. Taken together, these findings not only prove an essential role of NF-κB-mediated airway inflammation, but also illustrate the importance of alternative signaling pathway and additional cell types in the airways, and the complicated interactions between them in dictating the pathophysiology of asthma.

## Conclusion

Our data demonstrate that a broader anti-inflammatory activity of As_2_O_3 _lies in the inhibition of NF-κB activation through induction of IκBα expression in the airways. Clinically, low dosage of As_2_O_3 _may have a potential benefit in treating patients with asthma, especially in those with steroid-dependent and -resistant asthma [8,13]. It is anticipated that specific inhibitors of NF-κB may be developed by modifying the poisonous group(s) of As_2_O_3 _and screen As_2_O_3 _analogues in the libraries of chemical compounds. Moreover, novel nondegradable IκBα mutant, namely super-repressor of NF-κB, may be achieved by completely deleting the phosphorylation sites of Ser32 and Ser36 residues [18,37]. This will offer promising strategies for future immunotherapy of asthma as well as the infectious, inflammatory, cancerous and autoimmune diseases associated with aberrant NF-κB activation [1,5,39-42].

## Abbreviations

AHR, Airway hyperresponsiveness; ANOVA, One-way analysis of variance; APL, Acute promyelocytic leukemia; As_2_O_3_, Arsenic trioxide; ATRA, All-trans retinoic acid; BALF, Bronchoalveolar lavage fluid; ECL, Enhanced chemiluminescence; EOS, Eosinophils; ECA, EOS chemotaxis assay; ELISA, Enzyme-linked immunosorbant assay; EMSA, Electrophoretic mobility shift assay; HRP, Horseradish peroxidase; IL, Interleukin; IκB, Inhibitor of NF-κB; NF-κB, Nuclear factor κB; OVA, Ovalbumin; PBS, Phosphate-buffered saline; RANTES, Regulated upon activation, normal T cell expressed and secreted; SEM, Standard error of the mean; SDS-PAGE, Sodium dodecyl sulfate polyacrylamide gel eletrophoresis; Th2, T-helper type 2.

## Competing interests

The author(s) declare that they have no competing interests.

## Authors' contributions

LFZ conceived and designed the study, carried out all experiments, analyzed the data, and drafted the manuscript. YZ participated in the animal experiments, BALF cell counts, ECA, and ELISA. XFC performed the EMSA and Western blot analysis. WPX conducted the airway physiology, lung histology, and partial data analysis. AHH gave helpful advice for data analysis and interpretation. KSY coordinated most of the experiments and advised on data analysis. All authors read and approved the final manuscript.
